# Pulmonary Hemorrhage: An Unusual Life-Threatening Presentation of Factor IX Deficiency in a Monochorionic-Diamniotic Twin Neonate

**DOI:** 10.7759/cureus.20352

**Published:** 2021-12-11

**Authors:** Lisa M Pace, Andrew Y Lee, Sfurti Nath, Neil B Alviedo

**Affiliations:** 1 Pediatrics, University of Florida College of Medicine – Jacksonville, Jacksonville, USA; 2 Pediatric Medicine, University of Florida Health Jacksonville, Jacksonville, USA; 3 Pediatrics/Neonatal Perinatal Medicine, University of Florida College of Medicine – Jacksonville, Jacksonville, USA; 4 Neonatology, University of Florida College of Medicine – Jacksonville, Jacksonville, USA

**Keywords:** christmas disease, mono-di twin gestation, pulmonary hemorrhage, bleeding disorder, pediatrics & neonatology, hemophilia b, factor ix deficiency

## Abstract

Pulmonary hemorrhage is a rare, life-threatening condition affecting premature infants. There is no single etiological explanation for it but some common denominators include the presence of extreme prematurity, respiratory distress syndrome, surfactant use, birth asphyxia, etc. Although the incidence of pulmonary hemorrhage in neonates may be small, it is associated with a high risk of mortality. Congenital bleeding disorders such as hemophilia are rare coagulation disorders that have been known to present in the early neonatal period with an increased tendency for bleeding after blood draws, circumcision, surgical interventions, intracranial hemorrhage, oral or mucosal bleeding, and very rarely as gastrointestinal hemorrhage. There are no reports so far in the published literature of hemophilia presenting as pulmonary hemorrhage in early life. We report an unusual primary presentation of hemophilia B in a premature, monochorionic-diamniotic twin with acute life-threatening pulmonary hemorrhage with no family history of bleeding disorders.

## Introduction

Pulmonary hemorrhage is a well-known, though rare, life-threatening complication in premature infants with respiratory distress syndrome. Respiratory deterioration secondary to severe pulmonary hemorrhage leads to high mortality of 50% and as a result, nearly 60% of these infants develop bronchopulmonary dysplasia and the need for prolonged respiratory support.

Factor IX deficiency, also known as hemophilia B/Christmas Disease, is one of the most common, serious, congenital coagulation factor deficiencies. Hemophilia B has an X-linked recessive inheritance pattern and is seen in 1:30,000 male births [1,2]. In addition to the genetic inheritance, there is a significant rate of de novo mutations, which can contribute to about 30% to 50% of the cases [1,2]. Classic laboratory studies show a prolonged activated partial thromboplastin time (aPTT) and a normal prothrombin time (PT) and bleeding time. The prolonged aPTT is due to a decrease in factor IX activity. Factor IX in the coagulation cascade is associated with platelet activation and the formation of thrombin, eventually leading to the development of a fibrin clot [3,4,5].

Routine laboratory tests obtained in diagnosing Factor IX deficiency include a coagulation profile and mixing studies. Mixing studies are performed by combining the plasma of the patient with suspected hemophilia with normal plasma. An aPTT that normalizes confirms factor deficiency. If the aPTT does not normalize during a mixing study, the presence of an inhibitor is highly suspected [6,7].

In neonates, bleeding events are typically seen following complications after delivery or with prolonged bleeding during medical procedures, such as circumcisions [5,8]. Once the diagnosis is made, disease severity is determined by the level of remaining factor activity and classified accordingly as mild, moderate, or severe. Mild hemophilia is defined by hemorrhage with 5% to 40% factor activity with trauma, while moderate hemophilia results in hemorrhage with 1% to 5% factor activity with trauma [2,8]. Severe hemophilia presents with spontaneous bleeding and less than 1% factor activity [2,8].

Individuals classified as severe hemophiliacs have an increased risk for spontaneous bleeding and intracranial hemorrhage and rarely can present with gastrointestinal hemorrhage. In this case report, we present a premature monochorionic-diamniotic (mono-di) twin with no significant family history of bleeding diathesis, who presented with acute pulmonary hemorrhage on day of life (DOF) 7, with clinical workup and management leading to a diagnosis of hemophilia B.

## Case presentation

Our patient is a Hispanic, preterm, male twin (B) of a set of mono-di twins, born at 29+3 weeks gestation to healthy, non-consanguineous parents. Mother is a gravida 6 and para 5 with 3 preterm pregnancies prior to this delivery. The infant was delivered breech with Appearance, Pulse, Grimace, Activity, and Respiration (APGAR) scores of 7 and 8 at one and five minutes, respectively. Birth weight was 1.26 kg (34th percentile), length 35 cm (8th percentile), HC 27 cm (36th percentile). Neonatal resuscitation was remarkable for the need for continuous positive airway pressure (CPAP) during resuscitation with a continued requirement of respiratory support and oxygen (CPAP 5 cms of water at FiO2 0.3%) in the neonatal ICU (NICU). Vitamin K was administered at birth. Respiratory support was escalated to non-invasive positive pressure ventilation (NIPPV) shortly after admission, but he was eventually weaned to 2L high-flow nasal cannula (HFNC) by DOL 6.

**Figure 1:**
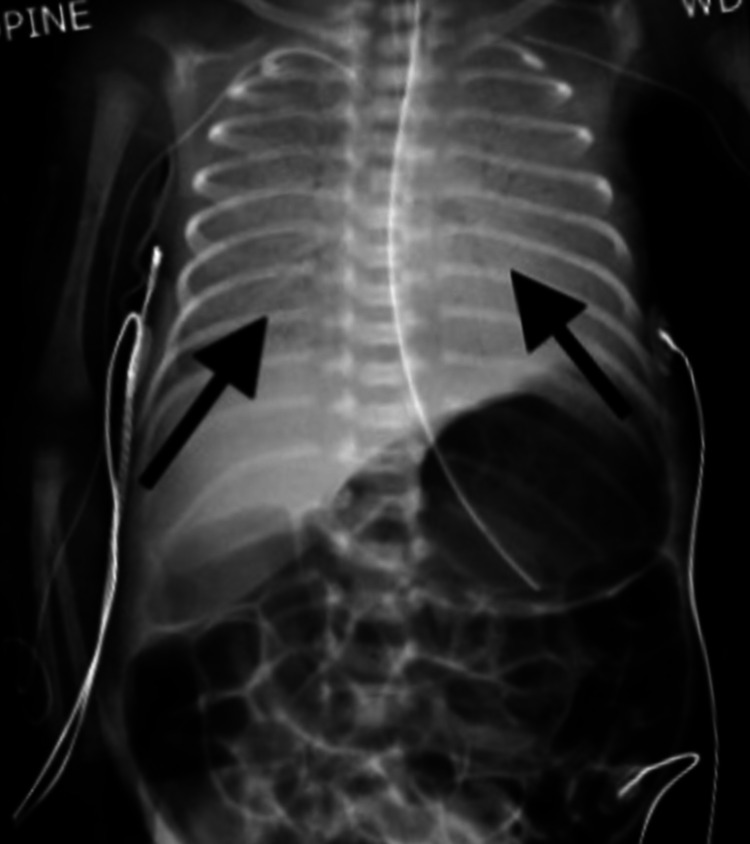
Chest/abdomen x-ray: AP view showing bilateral hazy opacities. No pleural effusion or pneumothorax was noted. AP: anteroposterior

On DOL 7, the neonate developed apneic episodes with lethargy and tachycardia (HR 180-200 beats per min). Sepsis workup was initiated including blood culture, complete blood count (CBC), and C-reactive protein. Antibiotics were initiated on the suspicion of sepsis. An abdominal x-ray was unremarkable and showed no bowel loop distension, pneumatosis, or free air. Due to recurrent apneic events with severe desaturation, it was decided by the clinical team to intubate the infant. Bloody secretions were obtained from the nares when the airway was suctioned in preparation for intubation. Additional blood-tinged secretions were noted in the oral cavity and at the glottic opening at the time of intubation. Cold saline lavage with epinephrine was emergently administered via the endotracheal tube (ET). An anteroposterior view of the chest x-ray (Figure 1) showed bilateral white-out of the lung fields suggestive of severe pulmonary edema and pulmonary hemorrhage.

Appropriate ventilator settings with a high positive end-expiratory pressure (PEEP) were initiated. He was then transfused with fresh frozen plasma (FFP) and packed red blood cells (pRBC), both at 20 ml/kg, followed by a dose of Lasix at 1mg/kg IV. The infant also received a second dose of vitamin K. Coagulation studies were obtained and showed a prolonged aPTT of 88 seconds and a markedly increased D-dimer of 2512. Fibrinogen, international normalized ratio (INR), and PT were all within acceptable neonatal ranges. CBC did not indicate signs of infection with no left shift. An echocardiogram was performed which showed a patent foramen ovale with left to right flow. Over the next three days, he received multiple transfusions of pRBC and FFP for continuing pulmonary hemorrhage and bright red blood suctioned from the ET tube. A complete abdominal ultrasound was obtained to rule out additional sources of bleeding. Daily coagulation studies showed persistently prolonged PTT with a maximum of 101 seconds. Head ultrasound performed on DOL 7 showed no evidence of intracranial hemorrhage. Pediatric hematology was consulted, and clotting factor studies were recommended. Factor VIII activity was normal at 96%. However, Factor IX activity was low at 35%, indicating mild hemophilia B. Factors XI and XII activities were within the normal range for age. Additionally, mixing studies were performed which indicated a factor deficiency - making the diagnosis of hemophilia B more definitive (Table 1).

**Table 1:** Mixing studies showing improvement of the aPTT after infant's plasma was combined with normal plasma aPTT: activated partial thromboplastin time

Component	Age (days)	Value	Ref. Range (seconds)
aPTT	N/A	48.2	23.1 - 32.5
aPTT 1:1 Normal Plasma(NP)	7	29.8	23.1 - 32.5
aPTT 1:1 NP Mix (60 min incubation)	7	37.2	23.1 - 32.5
aPTT 1:1 NP (incubation. Mix control)	7	32.6	23.1 - 32.5

**Figure 2:**
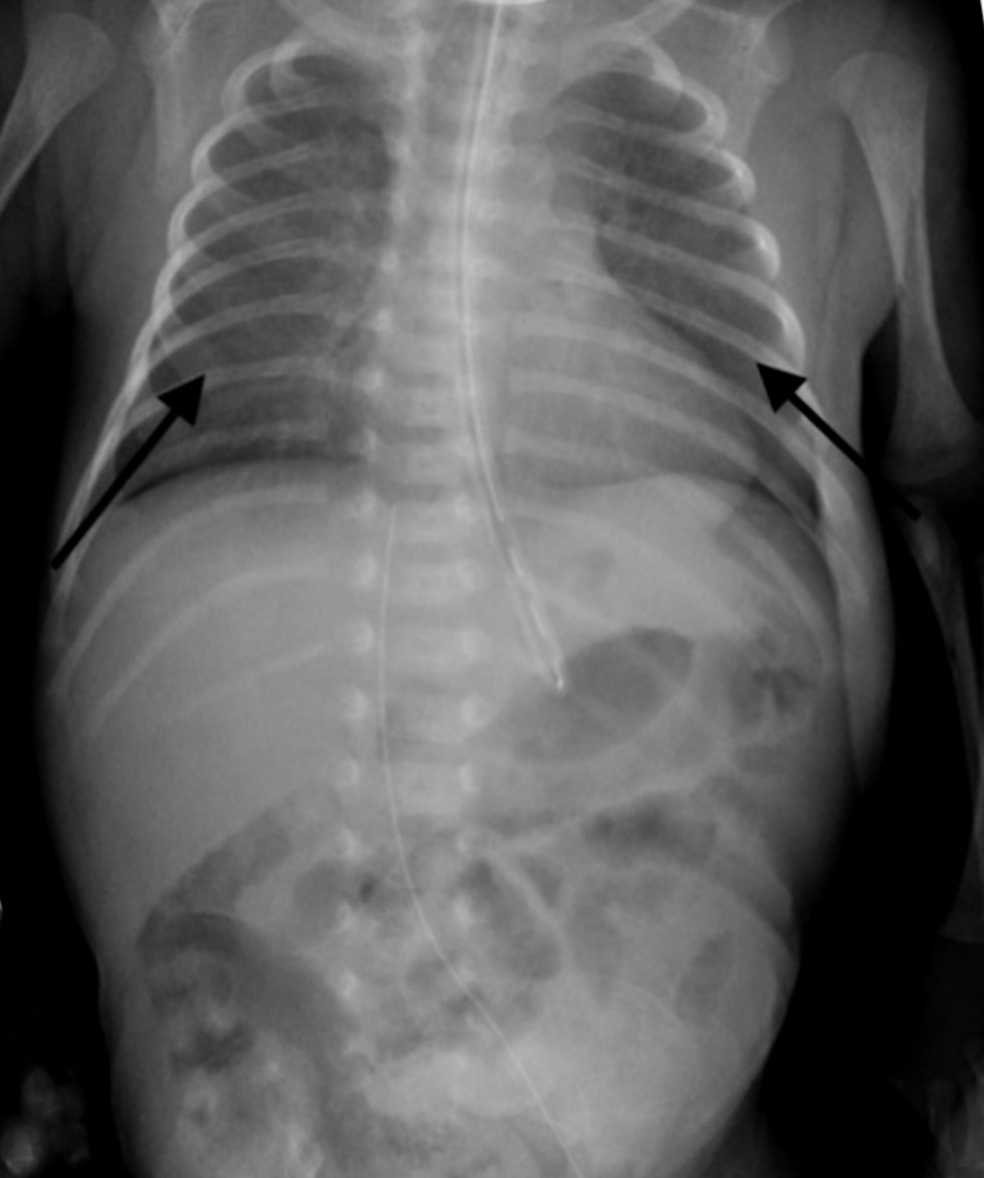
Post-extubation chest/abdomen x-ray: AP view showing significantly improved aeration of both lungs AP: anteroposterior

The infant was consequently treated with several doses of recombinant factor IX concentrate injection at 90 units given intravenously. Clinical improvement was noted with no further episodes of pulmonary hemorrhages or bleeding from any other sites (Figure 2). He was successfully extubated on DOL 18 to room air.

The sibling, Twin A, also underwent a workup for hemophilia B. His aPTT was slightly prolonged at 50.5 seconds and factor IX activity was 23% - lower than Twin B. Twin A, however, had an uncomplicated postnatal course and did not show any signs of bleeding diathesis. Both twins remain in the NICU, with plans to repeat factor IX levels at one month of age and follow up for genetics consultation.

## Discussion

Pulmonary hemorrhage is a severe, life-threatening condition that can be seen in early preterm neonates with very low birth weight (typically <1500 grams) [3,6]. Although the source of pulmonary hemorrhage is often unknown, there are several causes that must be ruled out including but not limited to, infection, trauma, and congenital heart disease [3,6,7]. In the case of our patient, the delivery method was an uncomplicated spontaneous vaginal delivery with no birth trauma/vacuum assistance or history of instrumentation. Infection was unlikely based on a normal CBC and negative blood culture results. He was also treated with broad-spectrum antibiotics for 48 hours with no improvement of symptoms. An echocardiogram ruled out congenital heart disease, including a patent ductus arteriosus (PDA), as a potential cause of pulmonary hemorrhage. The hematologic workup led to the diagnosis of coagulopathy in the form of factor IX deficiency. In many instances of clotting factor deficiency, including hemophilia A and B, the initial presenting symptom is intracranial hemorrhage [5] or excessive bleeding following circumcision, blood draws, or any surgical procedures and in some rare instances, gastrointestinal hemorrhage has been described. This case of hemophilia B, presenting with severe pulmonary hemorrhage has, to our knowledge, never been reported in the literature thus far.

The standard treatment for hemophilia involves the replacement of the deficient coagulation factor with a recombinant factor [5]. In acute settings, the management of hemophilic bleeding in newborns involves the administration of missing factors, either in the form of blood products (FFP) or with recombinant factors [5]. These factors can also be given prophylactically in the absence of overt signs of bleeding and are used as a lifelong treatment to prevent spontaneous bleeding for patients with hemophilia [5].

## Conclusions

We report a rare presentation of hemophilia B in a neonate with spontaneous bilateral pulmonary hemorrhage. In addition to reports of newborns with hemophilia presenting with bleeding after circumcision, spontaneous splenic rupture, bleeding from the umbilical stump, cephalohematoma, and intracranial hemorrhage, knowledge of rare manifestations of the disease can help the clinician in initiating early treatment. Overall, the management of hemophilia remains the same with factor replacement and assistance from a hematology team.

Hemophilia is a rare bleeding disorder with varied clinical presentation in the neonatal period. Knowledge of the different presentations and establishing early diagnosis at birth can be beneficial in initiating timely management and limiting morbidity and mortality associated with the condition.
